# Velocity-Dependent Eccentric Knee-Flexion Isokinetic Assessment in Elite Professional Soccer Players: Reliability, Inter-Limb Asymmetry and Mechanical Characteristics

**DOI:** 10.3390/jfmk11020184

**Published:** 2026-04-30

**Authors:** Francisco Javier Nuñez, Marco Carletta, Gloria Picco, Reyes Adorna, Juan Luis Nuñez-González, Luis Suarez-Arrones

**Affiliations:** 1 Physical Performance & Sports Research, Universidad Pablo de Olavide, 41013 Seville, Spain; ljsuamor@upo.es; 2 Performance and Health Department, FC Lugano, 6900 Lugano, Switzerland; carletta.marco1@gmail.com; 3 Centro dello Sport—Ars Medica, 6929 Lugano, Switzerland; picco.gloria@gmail.com; 4 Ministry of Educational Development and Vocational Training, Junta de Andalucía, 41092 Seville, Spain; reyadoblanc@gmail.com; 5 Department of Communication and Education, Universidad Loyola Andalucía, 41704 Seville, Spain; jlnunez@uloyola.es

**Keywords:** isokinetic dynamometry, eccentric knee flexion strength, angular velocity, reliability, professional soccer

## Abstract

**Objective:** The primary aim of this study was to compare eccentric knee flexion isokinetic performance at two commonly used angular velocities (60°·s^−1^ and 180°·s^−1^) between dominant and non-dominant limbs in healthy professional soccer players through the analysis of peak torque, mean peak torque, angle of peak torque, total work, and rate-of-torque-development-related variables. The secondary aim was to describe concentric knee extension and knee flexion strength variables assessed at 60°·s^−1^. **Methods:** Forty male professional soccer players performed concentric knee flexion–extension testing at 60°·s^−1^ and eccentric knee flexion testing at 60°·s^−1^ and 180°·s^−1^ using an isokinetic dynamometer. Peak torque (PT), mean peak torque (MPT), angle of peak torque (APT), total work (TW), and hamstrings: quadriceps ratios (H:Q ratios) were analyzed for dominant and non-dominant limbs. Inter-limb differences, repetition effects, and reliability indices were calculated. **Results:** No significant inter-limb differences were observed for most variables (trivial–small effect sizes), except for higher eccentric TW at 180°·s^−1^ in the dominant limb (*p* = 0.009). Eccentric PT and MPT decreased at higher velocities in both dominant (*p* = 0.002 and *p* < 0.001, respectively) and non-dominant (*p* = 0.008 and *p* < 0.001, respectively) limbs, while APT shifted toward more flexed knee angles (*p* < 0.001). Reliability was good to excellent (ICC = 0.81–0.87), with low measurement error. **Conclusion:** Eccentric knee flexion assessment at 60°·s^−1^ and 180°·s^−1^ angular velocities provided different results in PT, MPT, and APT for the same group of players, supporting the use of more than one eccentric test for obtaining information about these variables in elite soccer.

## 1. Introduction

Assessing the isokinetic strength of the quadriceps and hamstring musculature is crucial for quantifying neuromuscular capacity in professional soccer players, as these muscle groups generate the high torques required for sprinting, deceleration, change of direction, and powerful kicking actions [1]. Isokinetic dynamometry provides an objective assessment of concentric and eccentric peak torque across a joint’s range of motion, facilitating the detection of interlimb asymmetries, strength deficits, and neuromuscular adaptations following training or rehabilitation [2]. Given the high incidence of non-contact hamstring strain injuries among elite soccer players, routine isokinetic screening is often incorporated into preseason assessments and return-to-play decision-making to guide injury-prevention strategies [3]. Isokinetic profiles also facilitate individualized load management by identifying neuromuscular deficits in peak torque, total work, and rate-dependent measures that may not be detected through field-based tests alone [4].

In professional soccer research, the most commonly reported isokinetic variables include concentric and eccentric peak torque (PT) during knee extension (quadriceps) and flexion (hamstrings), measured at multiple angular velocities—frequently 60°/s, 180°/s, and higher speeds (e.g., 300°/s)—which together characterize both maximal strength and force–velocity behavior [5]. Conventional H:Q ratios (concentric hamstrings–concentric quadriceps) and functional ratios (eccentric knee flexions–concentric quadriceps) are widely calculated to assess the balance between agonist and antagonist muscle groups, while additional indices such as angle-specific ratios, bilateral strength asymmetry, and total work (TW) have been increasingly used to better reflect the rapid and dynamic demands of match play [6]. Normative data and systematic reviews indicate that conventional H:Q ratios typically range around ~50–70% at slower test velocities and that functional ratios can substantially increase when assessed at mixed velocities or eccentric-dominant actions, emphasizing the importance of velocity-specific reference values [6].

The relationship between isokinetic strength metrics and both performance outcomes and injury risk in elite soccer is multifaceted and cannot be fully captured by a single mechanical variable or testing condition. Greater quadriceps and hamstring peak torque values have been positively associated with sprinting, jumping, and change-of-direction performance, emphasizing the role of maximal force production during key match actions [5]. In addition, prospective studies indicate that reduced eccentric knee flexion strength and lower functional H:Q ratios are linked to an increased risk of hamstring strain injury, although effect sizes are moderate and many injured players do not present clear preseason strength deficits, limiting the predictive value of isolated peak-based measures [2,3]. This has led to increasing interest in angle-specific analyses, as peak torque and conventional H:Q ratios may fail to reflect torque balance across the full range of motion or during the early phases of force production, particularly at long muscle lengths where hamstring injuries typically occur [5,6]. From a methodological perspective, isokinetic protocols are generally standardized to ensure reliable estimation of peak torque and ratios, with consensus and reliability studies suggesting that 3–5 maximal repetitions at slower velocities (e.g., 60°·s^−1^) and 4–5 repetitions at higher velocities (e.g., 180°·s^−1^) are sufficient to achieve stable torque values while minimizing fatigue [7,8]. However, soccer-specific testing batteries often now include multiple velocities, contraction modes, and repetition schemes to derive additional variables such as total work and angle-specific torque, which may reflect distinct neuromuscular capacities relevant to performance and injury prevention [7]. Despite the widespread use of isokinetic testing to assess hamstring function in soccer players, important methodological aspects remain insufficiently understood. In particular, it remains unclear whether eccentric hamstring performance can be adequately characterized using a single testing velocity or whether assessments at different angular velocities provide complementary and non-redundant information. This issue is especially relevant given current recommendations encouraging multi-variable and multi-velocity profiling of neuromuscular function, while the extent to which different velocities capture distinct aspects of eccentric function remains unknown in elite soccer players. Furthermore, there is limited evidence comparing how multiple outcome variables (e.g., peak torque, angle-specific measures, and rate-of-torque-development-related metrics) behave across velocities and between limbs and whether they provide overlapping or unique information.

Therefore, the primary aim of this study was to compare eccentric knee flexion isokinetic performance at two commonly used angular velocities (60°·s^−1^ and 180°·s^−1^) between dominant and non-dominant limbs in healthy professional soccer players through the analysis of peak torque, mean peak torque, angle of peak torque, and total work variables. The secondary aim was to describe concentric knee extension and knee flexion strength variables assessed at 60°·s^−1^.

## 2. Materials and Methods

### 2.1. Study Design

A cross-sectional observational study design was used to examine concentric and eccentric isokinetic knee strength characteristics in professional soccer players. All participants completed a single testing session during which concentric knee extension and flexion were assessed at 60°·s^−1^, followed by eccentric knee flexion testing at 60°·s^−1^ and 180°·s^−1^. Five maximal repetitions were performed for each testing condition to allow the assessment of peak torque consistency, inter-limb comparisons, and the development of intra-session reliability indices. The selection of five repetitions represents a compromise between ensuring measurement reliability and minimizing fatigue and is consistent with established protocols in the literature [1,3,8].

### 2.2. Participants

Forty male professional soccer players (23.7 ± 3.5 years; 78.5 ± 6.3 kg; 182.1 ± 7.5 cm; 47.9 ± 5.8 mm (sum of 6 skinfolds)) were recruited from a Swiss first-division soccer club competing in the UEFA Conference League during 2023–2024, 2024–2025, and 2025–2026 seasons. Exclusion criteria were lack of full availability for regular participation in organized training and matches; a lower-limb injury in the last 6 weeks; a history of lower-limb surgery in the previous 12 months; current pain or symptoms preventing maximal testing; and any medical conditions contraindicating high-intensity neuromuscular testing. All participants provided written informed consent. The study was conducted according to the Declaration of Helsinki (2013), and the protocol was fully approved by the Virgen Macarena and Virgen del Rocio University Hospitals ethics committee (0398-N-17; approval date: 15 November 2017; Seville) before recruitment.

### 2.3. Procedures

Isokinetic testing was conducted using a Genu Plus isokinetic dynamometer (Easytech Srl, Florence, Italy). Prior to testing, players were instructed to refrain from strenuous exercise for ≥24 h, avoid caffeine for ≥12 h, and maintain consistent hydration and nutrition across sessions. All players were familiar with the isokinetic test. Before the isokinetic test session, the participants did a standardized warm-up (5 min cycling, dynamic mobility, and two sets of submaximal practice contractions). Participants were seated with ~85–90° hip flexion and were stabilized using pelvic, trunk, and thigh straps. Dynamometer alignment was adjusted to match the lateral femoral epicondyle. Prior to initiating the isokinetic protocol, the participant’s leg was weighted for gravitational error torque in accordance with the manufacturer’s procedures to account for its effect on torque. The dynamometer axis was carefully aligned with the knee joint center. The range of motion was standardized to 80°, spanning from 90° to 10° of knee flexion, with 0° defined as full knee extension. The same assessor performed all positioning procedures.

Concentric knee extension (quadriceps) and knee flexion (hamstrings) were assessed using a 60°·s^−1^: 5 maximal repetitions standardized protocol. Eccentric knee flexion (hamstrings) was assessed at 60°·s^−1^ and 180°·s^−1^: 5 maximal repetitions of standardized protocols. A rest period of 3 min was provided between tests. For each set, all repetitions were considered for analysis. Participants were verbally encouraged to perform maximal efforts. Trials were considered invalid and excluded from analysis if any of the following occurred: (i) failure to reach or maintain the preset angular velocity, (ii) incomplete execution of the predefined range of motion, (iii) evident submaximal effort or inconsistent torque production, (iv) technical or recording errors, or (v) interruption due to pain or discomfort. Invalid trials were removed.

The following variables were measured for dominant and non-dominant legs and used for analysis: peak torque (PT: highest torque value obtained in each set) (Nm); mean peak torque (MPT: mean of the five highest peak torque values per set) (Nm); angle peak torque (APT: the joint angle (°) at which peak torque occurred during the movement). Joint angles were recorded at the angle of peak torque during the knee extension (APT_EXT-CON60_) and knee flexion (APT_FLE-CON60_) during the concentric test at 60°·s^−1^, and during knee flexion in the eccentric test at 60°·s^−1^ (APT_FLE-ECC60_) and 180°·s^−1^ (APT_FLE-ECC180_). In addition, total work (TW) (J) was calculated as the sum of torques across the full range of motion for all repetitions during the knee extension and flexion (TW_FLE-CON60_) during concentric (60°·s^−1^) (TW_EXT-CON60_ and TW_FLE-CON60_, respectively) tests and only flexion during eccentric (60°·s^−1^ and 180°·s^−1^) tests (TW_FLE-ECC60_ and TW_FLE-ECC180_, respectively). The conventional H:Q ratio was calculated (concentric PT during knee flexion–concentric PT during knee extension at 60°·s^−1^). The functional H:Q ratio was calculated (eccentric PT during knee flexion–concentric PT during knee extension at 60°·s^−1^).

### 2.4. Statistical Analysis

All statistical analyses were performed using JASP, version 0.96.0 (JASP Team, University of Amsterdam, Amsterdam, The Netherlands). Data distribution was assessed for normality using the Shapiro–Wilk test. Descriptive statistics are presented as mean ± standard deviation (SD). Differences between the dominant and non-dominant limbs were analyzed using paired-samples *t*-tests for each concentric and eccentric variable. Effect sizes were calculated using Cohen’s d and interpreted as trivial (<0.20), small (0.20–0.49), moderate (0.50–0.79), or large (≥0.80), according to established thresholds. To examine potential differences across the five repetitions, a one-way repeated-measures analysis of variance (ANOVA) was conducted separately for each variable, limb, and testing condition. The assumption of sphericity was evaluated using Mauchly’s test, and when violated, the Greenhouse–Geisser correction was applied to adjust the degrees of freedom. When significant main effects were detected, post hoc pairwise comparisons with Bonferroni adjustment were applied. Relative reliability was assessed using the intraclass correlation coefficient (ICC) with 95% confidence intervals (CIs), calculated using a two-way random-effects model with absolute agreement (ICC 2,1). Absolute reliability was evaluated using the standard error of measurement (SEM), calculated as follows:

SEM = SD × √(1 − ICC)

SEM was also expressed as a percentage of the mean (SEM%). The minimal detectable change at the 95% confidence level (MDC_95_) was calculated as follows:

MDC_95_ = SEM × 1.96 × √2

MDC_95_ values were reported in absolute units (Nm) to facilitate clinical interpretation. For all inferential analyses (e.g., repeated-measures ANOVA), the level of statistical significance was set at *p* < 0.05. Reliability metrics (ICC, SEM, and MDC_95_) are descriptive and not subject to hypothesis testing or multiple-comparison correction.

## 3. Results

The values obtained for the dominant and non-dominant legs from the concentric isokinetic test at 60°·s^−1^ for both knee flexion and extension, and from the eccentric isokinetic tests at 60°·s^−1^ and 180°·s^−1^ for knee flexion, are presented in Table 1. No statistically significant differences were observed between the dominant and non-dominant limbs for any concentric or eccentric variable or for the conventional and functional H:Q ratios (*p* > 0.05), with effect sizes ranging from trivial to small, except for TW_FLEX-ECC180_, where the dominant leg generated significantly greater total work than the non-dominant leg (+5%; small ES).

**Figure 1:**
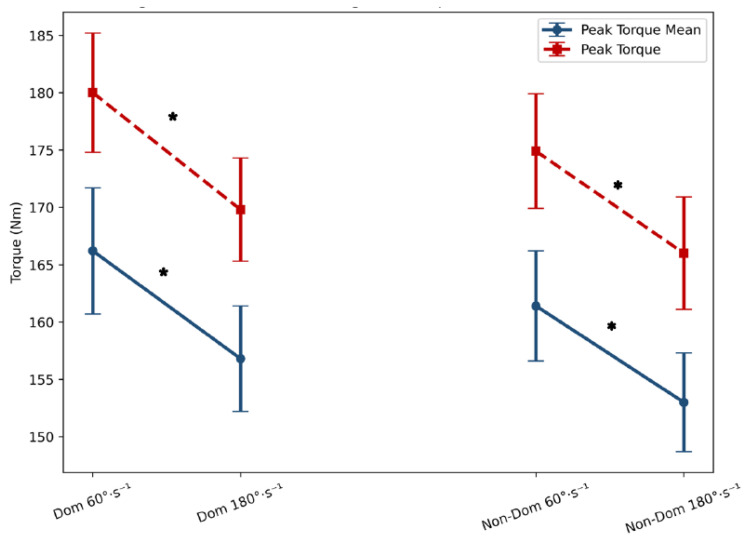
Comparison of eccentric isokinetic variables (MPT and PT) at 60°·s^−1^ and 180°·s^−1^ for dominant and non-dominant limbs. Data are presented as mean ± standard error (SE). * Significant differences between 60°·s^−1^ and 180°·s^−1^ eccentric isokinetic tests.

**Figure 2:**
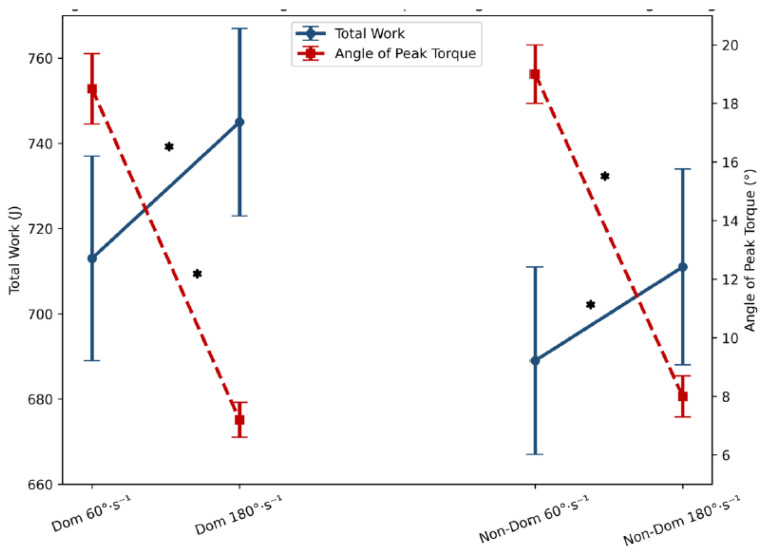
Comparison of angle of peak torque and total work during an isokinetic eccentric test at 60°·s^−1^ and 180°·s^−1^ in dominant and non-dominant limbs. Data are presented as mean ± standard error (SE). * Significant differences between 60°·s^−1^ and 180°·s^−1^ eccentric isokinetic tests.

The MPT and PT values were consistently and significantly higher at 60°·s^−1^ compared to 180°·s^−1^ (see Figure 1) in both dominant (*p* = 0.002, ES = 0.29 and *p* < 0.001, ES = 0.32, respectively) and non-dominant limbs (*p* = 0.008, ES = 0.29 and *p* < 0.001, ES = 0.72, respectively). Similarly, the APT occurred at significantly greater knee flexion angles at 60°·s^−1^ compared to 180°·s^−1^ (see Figure 2) for both limbs (*p* < 0.001, ES = 1.25–1.30), reflecting a velocity-dependent shift in torque production. Conversely, TW tended to be greater at 180°·s^−1^ (see Figure 2), particularly in the dominant limb (*p* = 0.041, ES = 0.21). Error bars indicated low variability, supporting the robustness of the measurements across conditions.

**Figure 3:**
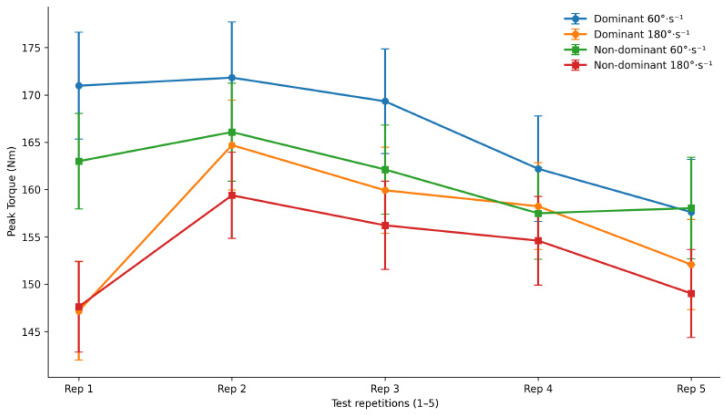
Peak torque values across five repetitions of the isokinetic test for dominant and non-dominant limbs at 60°·s^−1^ and 180°·s^−1^. Data are presented as mean ± standard error (SE). The *x*-axis represents the five consecutive repetitions performed within the test.

Figure 3 illustrates the evolution of PT values across the five consecutive repetitions performed during the isokinetic test for both limbs and angular velocities. For both dominant and non-dominant legs, PT values remained relatively stable across repetitions with no significant increasing or decreasing trends (*p* > 0.05), indicating consistent torque production throughout the test.

The reliability analysis demonstrated good to excellent relative reliability for eccentric PT measurements at both angular velocities. Intraclass correlation coefficients ranged from 0.805 to 0.871 for the dominant limb and from 0.816 to 0.818 for the non-dominant limb across 60°·s^−1^ and 180°·s^−1^ conditions (see Table 2). Absolute reliability was high, with SEM values ranging from 2.09 to 2.53 Nm, corresponding to 1.36–1.52% of the mean values. The minimal detectable change at the 95% confidence level ranged between 5.79 and 7.00 Nm, indicating that changes exceeding these thresholds can be interpreted as real changes beyond measurement error (see Table 2).

## 4. Discussion

The aims of this study were to describe concentric knee extension and flexion strength at 60°·s^−1^ and to compare eccentric knee flexion isokinetic performance at two commonly used angular velocities (60°·s^−1^ and 180°·s^−1^) in the dominant and non-dominant limbs of male professional soccer players. The main findings were as follows: (a) Professional soccer players exhibited a high degree of bilateral symmetry in both concentric and eccentric knee strength variables, with no meaningful differences between limbs. (b) Eccentric knee flexion performance exhibited clear velocity-dependent differences, with reductions in peak torque and systematic shifts in the angle of peak torque at higher angular velocities. (c) The eccentric isokinetic protocol showed high within-test consistency and good to excellent reliability, supporting its application for performance monitoring and injury-prevention screening.

No statistically significant differences were observed between dominant and non-dominant limbs for concentric knee extension and knee flexion PT, eccentric knee flexion strength at 60°·s^−1^ and 180°·s^−1^ PT, APT, or conventional and functional H:Q ratios. Effect sizes ranging from trivial to small indicated limited practical relevance. These findings align with previous investigations in professional soccer players showing minimal bilateral asymmetries when isokinetic testing is conducted under standardized laboratory conditions [3,8,9]. Croisier et al. [9] and van Dyk et al. [3] reported that absolute side-to-side differences in peak torque and H:Q ratios in professional players are generally small and clinically relevant only when exceeding established asymmetry thresholds. The conventional H:Q ratio (~0.73) and functional H:Q ratio (~0.85) observed in the present cohort are consistent with normative values reported in professional soccer players [8,10,11], reinforcing the notion that balanced agonist–antagonist strength profiles are characteristic of high-level players. The absence of limb differences agrees with previous work indicating that explosive torque production is highly symmetrical in trained soccer players [12], likely reflecting bilateral exposure to sprinting and high-intensity actions. The only variable showing a significant inter-limb difference was total work during eccentric knee flexion testing at 180°·s^−1^, with the dominant limb producing larger amounts (moderate effect size). This isolated finding may reflect subtle neuromuscular or coordination-related adaptations associated with preferred kicking limb usage, as suggested in earlier studies examining eccentric hamstring loading during soccer-specific actions [11,13]. However, given the preservation of symmetry in peak torque and H:Q ratios, this difference should be interpreted cautiously. Overall, the present findings reinforce the prevailing evidence that bilateral symmetry is typical in professional soccer players, but more studies are necessary to analyze the reason for a possible asymmetry in the total work variable.

The absolute values of concentric and eccentric PT, MPT, APT, and TW reported in this study are consistent with those previously described in uninjured professional soccer players. Concentric knee extension PT values (~210–215 Nm), knee flexion PT (~150 Nm) at 60°·s^−1^, and eccentric knee flexion PT at 60°·s^−1^ and 180°·s^−1^ (~170–180 Nm) fell within established normative ranges for athletes in general and footballers in particular [9,10,11,14]. APT values were also comparable to previous reports, with knee extension PT occurring at mid-to-late ranges and at relatively shorter muscle lengths during knee flexion through concentric actions [10,12].

With respect to TW, our results were generally symmetrical between limbs across concentric and eccentric conditions, except for eccentric knee flexion at 180°·s^−1^. In uninjured professional soccer players, TW has been shown to be relatively symmetrical between limbs and closely associated with repeated force production capacity and fatigue resistance during dynamic tasks [10,13]. In contrast, reduced total work (particularly during eccentric knee flexion testing) has been consistently reported in players with a history of hamstring injury, even after return to play, suggesting persistent impairments in the ability to sustain force output across the range of motion [3,9]. The preservation of both PT and MPT and TW values in the present cohort therefore supports the interpretation that these players could exhibit a robust and fatigue-resistant strength profile, characteristic of healthy elite soccer players. Collectively, these findings reinforce the value of combining torque- and work-based variables to achieve a more comprehensive characterization of knee flexion–extension function, improving the sensitivity of isokinetic assessments for performance monitoring.

The present study provides a comprehensive comparison of eccentric knee flexion isokinetic performance at two commonly used angular velocities (60°·s^−1^ and 180°·s^−1^) in professional soccer players, highlighting clear velocity-dependent differences across several key mechanical variables. The observed reductions in PT and MPT at higher angular velocities are consistent with the force–velocity relationship observed during eccentric hamstring actions [8,9,15]. A key finding of the present study was the systematic shift in the APT toward more flexed knee positions at the higher angular velocity (180°·s^−1^). This behavior has been consistently described in the literature and is thought to reflect alterations in muscle–tendon interaction and neuromuscular control as contraction speed increases [15,16,17]. In this context, the reduced APT observed at 180°·s^−1^ may indicate a diminished capacity to produce peak force at longer muscle lengths, a factor of particular relevance for injury prevention screening [11,18]. Interestingly, TW tended to be higher at 180°·s^−1^, especially in the dominant limb. This finding suggests that despite lower peak torque values, players were able to maintain force production across the entire range of motion at higher velocities. Similar results have been reported in team sport players and are thought to reflect sport-specific adaptations to repeated high-speed actions, such as sprinting and change of direction [10,19]. Accordingly, TW has been proposed as a complementary metric to PT, as it captures the capacity to sustain eccentric force rather than maximal output alone [14]. These findings reinforce the importance of assessing eccentric knee flexion across multiple angular velocities. While testing at 60°·s^−1^ provides valuable information regarding maximal force-generating capacity, higher velocities such as 180°·s^−1^ may complement this assessment by identifying mechanically and clinically relevant characteristics related to sprint-specific demands [16,19,20,21].

The stable PT values observed across five consecutive repetitions, regardless of limb dominance or angular velocity, indicate a high level of within-test consistency and suggest that fatigue or learning effects did not meaningfully influence torque production during the protocol [8]. This finding supports the appropriateness of the selected number of repetitions for eccentric knee flexion assessment, aligning with previous studies that recommend multiple repetitions to ensure reliable torque estimation while minimizing neuromuscular fatigue during isokinetic testing [20,21]. The good to excellent intraclass correlation coefficients obtained across both velocities and limbs further confirm the robustness of the protocol for assessing eccentric peak torque in professional soccer players [3,12]. Notably, the very low SEM values (1.36–1.52% of the mean) indicate high absolute reliability, reflecting minimal measurement error and reinforcing the precision of the dynamometer–protocol combination [20,22]. From a practical perspective, the relatively small MDC_95_ values (≈6–7 Nm) underscore the ability of the test to detect meaningful neuromuscular changes over time [3,23]. Collectively, these findings align with previous reliability studies in elite athletic populations and highlight the clinical and performance-related utility of eccentric isokinetic testing when appropriate methodological controls are applied [1,6].

The cross-sectional design of the present study limits the ability to establish causal relationships between eccentric knee flexion isokinetic variables and performance outcomes or injury risk. While the observed velocity-dependent differences provide valuable descriptive and reliability information, longitudinal designs are required to determine whether these mechanical characteristics are predictive of future hamstring strain injuries or meaningful changes in on-field performance. In addition, although two commonly used eccentric angular velocities (60°·s^−1^ and 180°·s^−1^) were assessed, higher velocities (e.g., ≥300°·s^−1^), which may more closely replicate the rapid muscle–tendon loading experienced during maximal sprinting, were not included and could offer further insight into high-speed neuromuscular function. Finally, the absence of complementary neuromuscular or structural assessments (such as electromyography, muscle architecture imaging, or muscle–tendon stiffness measurements) limited the mechanistic interpretation of the velocity-related changes observed in PT, APT, and TW, as the underlying neural and morphological contributors could not be directly examined. Additionally, the observed standard error of measurement (SEM) values were relatively low. While this likely reflects the standardized testing protocol, participant familiarization, and the use of a homogeneous sample of professional soccer players, it is also possible that methodological factors—such as the limited number of repetitions per condition—contributed to reduced variability. Therefore, the reported SEM values may represent both true measurement precision and potential methodological bias. Future studies should consider evaluating reliability across multiple variables and larger, more heterogeneous samples to better characterize the generalizability of these findings.

## 5. Conclusions

This study provides a comprehensive characterization of concentric and eccentric isokinetic knee strength in professional soccer players, showing that bilateral symmetry and normative strength profiles are characteristics of healthy elite populations. Concentric testing at 60°·s^−1^ effectively captured maximal strength capacity, whereas eccentric knee flexion assessments revealed marked velocity-dependent differences in mechanical behavior. Specifically, higher testing velocities were associated with reductions in peak torque, shifts in the angle of peak torque toward more flexed knee positions, and possible alterations in total work that are not evident at slower velocities alone. The high reliability and low measurement error observed across all testing conditions further support the robustness of the applied protocol. Collectively, these findings indicate that a single eccentric isokinetic test is insufficient to fully characterize hamstring function in professional soccer players. Accordingly, the integration of multiple eccentric velocities and complementary variables such as PT, MPT, TW, and angle-specific measures is recommended to enhance the sensitivity of isokinetic assessment in footballers.

## 6. Practical Applications

The high inter-limb symmetry observed suggests that small side-to-side differences in peak torque or H:Q ratios may have clinical relevance in healthy professional soccer players.

The present findings suggest that eccentric knee flexion strength testing at both 60°·s^−1^ and 180°·s^−1^ reflects distinct neuromuscular capacities. While 60°·s^−1^ provides insight into maximal force-generating capacity, higher angular velocities more closely reflect sprint-specific demands and may reveal mechanical behaviors (e.g., reduced peak torque or shifts in the angle of peak torque) that are not evident at slower speeds.

The excellent reliability and low SEM values indicate that eccentric knee flexion testing using five repetitions is suitable for tracking meaningful neuromuscular changes over time.

## Figures and Tables

**Table 1:** Comparison of the values obtained in the concentric isokinetic test at 60°·s^−1^, and eccentric at 60°·s^−1^ and 180°·s^−1^ for the dominant and non-dominant leg (mean ± SD).

	Dominant	Non-Dominant	*p*	Cohen’s d
MPT_EXT-CON60_ (Nm)	198.7 ± 31.4	201.7 ± 31.8	0.467	−0.12 (−0.43; 0.20)
MPT_FLE-CON60_ (Nm)	139.4 ± 19.51	143.6 ± 24	0.086	−0.28 (−0.59; 0.04)
PT_EXT-CON60_ (Nm)	213.3 ± 33.1	210 ± 32.3	0.419	0.13 (−0.18; 0.44)
PT_FLE-CON60_ (Nm)	153.3 ± 24.4	149.9 ± 19.9	0.174	0.22 (−0.1; 0.53)
APT_EXT-CON60_ (°)	58.5 ± 6.5	59.1 ± 6.2	0.520	−0.1 (−0.41; 0.21)
APT_FLE-CON60_ (°)	29.2 ± 11	28.0 ± 9.7	0.446	0.12 (−0.19; 0.43)
Conventional H:Q Ratio	0.7 ± 0.1	0.73 ± 0.2	0.947	−0.01 (−0.32; 0.3)
Functional H:Q Ratio	0.85 ± 10.9	0.8 ± 0.2	0.888	0.02 (−0.29; 0.27)
TW_EXT-CON60_ (J)	935.9 ± 184.1	931.8 ± 154.6	0.833	0.03 (−0.28; 0.34)
TW_FLE-CON60_ (J)	682.7 ± 155.6	664.9 ± 145.21	0.241	0.19 (−0.13; 0.5)
MPT_FLE-ECC60_ (Nm)	166.4 ± 34.2	161.4 ± 29.5	0.128	0.25 (−0.07; 0.56)
PT_FLE-ECC60_ (Nm)	180.2 ± 34	174.8 ± 31.6	0.133	0.24 (−0.07; 0.56)
APT_FLE-ECC60_ (°)	17.9 ± 10.4	19 ± 9.6	0.431	−0.13 (−0.44; 0.19)
TW _FLE-ECC60_ (J)	713.4 ± 150.1	689.2 ± 137.7	0.131	0.24 (−0.07; 0.56)
MPT_FLE-ECC180_ (Nm)	156.8 ± 28.3	152.9 ± 28	0.171	0.22 (−0.1; 0.53)
PT_FLE-ECC180_ (Nm)	169.7 ± 28.5	166 ± 29	0.174	0.22 (−0.1; 0.53)
APT_FLE-ECC180_ (°)	17.1 ± 5	18.4 ± 4.9	0.134	−0.24 (−0.56; 0.07)
TW _FLE-ECC180_ (J)	744.9 ± 137.9	711.5 ± 143.9	0.009 *	0.43 (0.11; 0.75)

MPT_EXT-CON60_: peak torque mean during extension concentric test; MPT_FLE-CON60_: peak torque mean during flexion concentric test; PT_EXT-CON60_: peak torque during extension concentric test; PT_FLE-CON60_: peak torque during flexion concentric test; APT_EXT-CON60_: angle peak torque during extension concentric test; APT_FLE-CON60_: angle peak torque during flexion concentric test; TW_EXT-CON60_: total work during extension concentric test; TW_FLE-CON60_: total work during flexion concentric test; MPT_FLE-ECC60_: peak torque mean during 60°·s^−1^ eccentric test; PT_FLE-ECC60_: peak torque during 60°·s^−1^ eccentric test; APT_FLE-ECC60_: angle peak torque during 60°·s^−1^ eccentric test; TW_FLE-ECC60_: total work during 60°·s^−1^ eccentric test; MPT_FLE-ECC180_: peak torque mean during 180°·s^−1^ eccentric test; PT_FLE-ECC180_: peak torque during 180°·s^−1^ eccentric test; APT_FLE-ECC180_: angle peak torque during 180°·s^−1^ eccentric test; TW_FLE-ECC180_: total work during 180°·s^−1^ eccentric test. * Significant differences between limbs.

**Table 2:** Reliability of eccentric peak torque measurements at 60°·s^−1^ and 180°·s^−1.^

	ICC (95% CI)	SEM (Nm)	SEM (%)	MDC_95_ (Nm)
60°·s^−1^ ECC Dominant	0.871 (0.807–0.921)	2.53	1.52	7.00
60°·s^−1^ ECC Non-dominant	0.818 (0.735–0.886)	2.24	1.39	6.20
180°·s^−1^ ECC Dominant	0.805 (0.718–0.878)	2.16	1.38	5.98
180°·s^−1^ ECC Non-dominant	0.816 (0.732–0.885)	2.09	1.36	5.79

## Data Availability

The original contributions presented in this study are included in the article. Further inquiries can be directed to the corresponding author.
